# SYRCLE’s risk of bias tool for animal studies

**DOI:** 10.1186/1471-2288-14-43

**Published:** 2014-03-26

**Authors:** Carlijn R Hooijmans, Maroeska M Rovers, Rob BM de Vries, Marlies Leenaars, Merel Ritskes-Hoitinga, Miranda W Langendam

**Affiliations:** 1 SYRCLE at Central Animal Laboratory, Radboud University Medical Center, Nijmegen, the Netherlands; 2 Centre of Evidence-based Surgery, Radboud University Medical Center, Nijmegen, the Netherlands; 3 Dutch Cochrane Centre, Academic Medical Center, University of Amsterdam, Amsterdam, the Netherlands

**Keywords:** Risk of bias, Methodological quality, Animal studies, Systematic reviews, Tool, Translational research

## Abstract

**Background:**

Systematic Reviews (SRs) of experimental animal studies are not yet common practice, but awareness of the merits of conducting such SRs is steadily increasing. As animal intervention studies differ from randomized clinical trials (RCT) in many aspects, the methodology for SRs of clinical trials needs to be adapted and optimized for animal intervention studies. The Cochrane Collaboration developed a Risk of Bias (RoB) tool to establish consistency and avoid discrepancies in assessing the methodological quality of RCTs. A similar initiative is warranted in the field of animal experimentation.

**Methods:**

We provide an RoB tool for animal intervention studies (SYRCLE’s RoB tool). This tool is based on the Cochrane RoB tool and has been adjusted for aspects of bias that play a specific role in animal intervention studies. To enhance transparency and applicability, we formulated signalling questions to facilitate judgment.

**Results:**

The resulting RoB tool for animal studies contains 10 entries. These entries are related to selection bias, performance bias, detection bias, attrition bias, reporting bias and other biases. Half these items are in agreement with the items in the Cochrane RoB tool. Most of the variations between the two tools are due to differences in design between RCTs and animal studies. Shortcomings in, or unfamiliarity with, specific aspects of experimental design of animal studies compared to clinical studies also play a role.

**Conclusions:**

SYRCLE’s RoB tool is an adapted version of the Cochrane RoB tool. Widespread adoption and implementation of this tool will facilitate and improve critical appraisal of evidence from animal studies. This may subsequently enhance the efficiency of translating animal research into clinical practice and increase awareness of the necessity of improving the methodological quality of animal studies.

## Background

The use of systematic reviews (SRs) for making evidenced-based decisions on healthcare is common practice in the clinical setting. Although most experimental animal studies aim to test safety and or efficacy of treatments to be used for human healthcare, summarizing the available evidence in an SR is far less common in the field of laboratory animal experiments. Fortunately, since an influential commentary was published in the Lancet (2002) [1], first setting out the scientific rationale for SRs of animal studies, awareness of the merits of SRs of experimental animal studies has been steadily increasing [2]. The methodology for conducting SRs of animal intervention studies is currently evolving but not yet as advanced as for clinical studies. In the clinical field, the randomized controlled trial (RCT) is considered the paradigm for evaluating the effectiveness of interventions. Animal intervention studies, like RCTs, are experimental studies, but they differ from RCTs in many respects [3] (Table 1, supporting information in Additional file 1). This means that some aspects of the systematic review process need to be adapted to the characteristics of animal intervention studies. In this paper, we focus on the methodology for assessing the risk of bias in animal intervention studies.

**Table 1:** Main differences between randomized clinical trials (RCTs) and animal intervention studies

**RCT**	**Animal intervention study**
Objective: demonstrating clinical efficacy	Objective: understanding disease mechanisms, suggesting intervention strategies(guiding clinical trials), examining potential efficacy, safety and toxicity of interventions
Disease naturally present	Disease often induced (with unclear/insufficient similarity to the human condition)
Timing of applying the intervention in relation to the disease onset is often heterogeneous	Intervention is often applied at a known time point in relation to the induced disease state
Often a heterogeneous group of patients (for example, lifestyle and co-morbidities)	Often a considerably homogeneous study population (e.g., comparable/controlled housing conditions and animal characteristics such as genetic backgrounds, gender, and presence of co-morbidities)
Sample size relatively large (compared to animal studies)**	Sample size relatively small (compared to RCTs) and sample size calculations often not reported
In general, relatively high internal validity because of randomization and blinding (compared to animal studies)**	In general, low internal validity (compared to RCTs)
	E.g., not yet standard practice to:
	-Randomize allocation of the animal to the intervention and control groups
	-Blind personnel and outcome assessors
Patients can be blinded for treatment in many situations.	Animals cannot and need not be blinded for treatment.
Relatively high external validity (extrapolation within one species)	Relatively low external validity (extrapolation between different species)
Relatively large teams involved	Relatively small teams involved
Intervention staffs are often different from outcome assessment staff.	One researcher is often responsible for treatment allocation and administration, outcome assessment and data analysis.
In general, no post-mortem data	In general, post-mortem material available
	Animals are often sacrificed at the end of the experiment.
Outcomes are often patient-relevant outcomes (compared to animal studies)	Outcomes are often surrogate outcomes, and still difficult to translate to the clinical setting even if similar to clinical outcomes
Clear guidelines for reporting and methodological quality [25]	Evolving guidelines for reporting and methodological quality [2,23,24]

**Additional file 1 provides some supportive information for this statement.

The differences described in this Table indicate general tendencies and may, therefore, not apply to all RCTs and animal intervention studies.

The extent to which an SR can draw reliable conclusions depends on the validity of the data and the results of the included studies [4-8]. Assessing the risk of bias of the individual studies, therefore, is a key feature of an SR. To assess the risk of bias of RCTs, the Cochrane Collaboration developed the Cochrane RoB Tool [9]. Such a general tool is not yet available for animal intervention studies. The checklists and scales currently used for assessing study validity of animal studies [10-14] vary greatly, are sometimes designed for a specific field (i.e., toxicology) and often assess reporting quality and internal and external validity simultaneously. We believe that, although it is important to asses all aspects of study quality in an SR, the assessment and interpretation of these aspects should be conducted separately. After all, the consequences of poor reporting, methodological quality and generalizability of the results are very different. Here, the SYstematic Review Centre for Laboratory animal Experimentation (SYRCLE) presents an RoB tool for animal intervention studies: SYRCLE’s RoB tool. This tool, based on the Cochrane Collaboration RoB Tool [9], aims to assess methodological quality and has been adapted to aspects of bias that play a role in animal experiments.

## Methods

### Development of SYRCLE’s RoB tool

The Cochrane RoB Tool was the starting-point for developing an RoB tool for experimental animal studies. The Cochrane RoB Tool assesses the risk of bias of RCTs and addresses the following types of biases: selection bias, performance bias, attrition bias, detection bias and reporting bias [9]. The items in the Cochrane RoB Tool that were directly applicable to animal experiments were adopted (Table 2: items 1, 3, 8, 9 and 10).

**Table 2:** SYRCLE’s tool for assessing risk of bias

**Item**	**Type of bias**	**Domain**	**Description of domain**	**Review authors judgment**
1	Selection bias	Sequence generation	Describe the methods used, if any, to generate the allocation sequence in sufficient detail to allow an assessment whether it should produce comparable groups.	Was the allocation sequence adequately generated and applied? (*)
2	Selection bias	Baseline characteristics	Describe all the possible prognostic factors or animal characteristics, if any, that are compared in order to judge whether or not intervention and control groups were similar at the start of the experiment.	Were the groups similar at baseline or were they adjusted for confounders in the analysis?
3	Selection bias	Allocation concealment	Describe the method used to conceal the allocation sequence in sufficient detail to determine whether intervention allocations could have been foreseen before or during enrolment.	Was the allocation adequately concealed? (*)
4	Performance bias	Random housing	Describe all measures used, if any, to house the animals randomly within the animal room.	Were the animals randomly housed during the experiment?
5	Performance bias	Blinding	Describe all measures used, if any, to blind trial caregivers and researchers from knowing which intervention each animal received. Provide any information relating to whether the intended blinding was effective.	Were the caregivers and/or investigators blinded from knowledge which intervention each animal received during the experiment?
6	Detection bias	Random outcome assessment	Describe whether or not animals were selected at random for outcome assessment, and which methods to select the animals, if any, were used.	Were animals selected at random for outcome assessment?
7	Detection bias	Blinding	Describe all measures used, if any, to blind outcome assessors from knowing which intervention each animal received. Provide any information relating to whether the intended blinding was effective.	Was the outcome assessor blinded?
8	Attrition bias	Incomplete outcome data	Describe the completeness of outcome data for each main outcome, including attrition and exclusions from the analysis. State whether attrition and exclusions were reported, the numbers in each intervention group (compared with total randomized animals), reasons for attrition or exclusions, and any re-inclusions in analyses for the review.	Were incomplete outcome data adequately addressed? (*)
9	Reporting bias	Selective outcome reporting	State how selective outcome reporting was examined and what was found.	Are reports of the study free of selective outcome reporting? (*)
10	Other	Other sources of bias	State any important concerns about bias not covered by other domains in the tool.	Was the study apparently free of other problems that could result in high risk of bias? (*)

*Items in agreement with the items in the Cochrane Risk of Bias tool.

To investigate which items in the tool might require adaptation, the differences between randomized clinical trials and animal intervention studies were set out (Table 1). Then we checked whether aspects of animal studies that differed from RCTs could cause bias in ways that had not yet been taken into account in the Cochrane RoB tool. Finally, the quality assessments of recent systematic reviews of experimental animal studies were examined to confirm that all aspects of internal validity had been taken into consideration in SYRCLE’s RoB tool.

To enhance transparency and applicability, we formulated signaling questions (as used in the QUADAS tool, a tool to assess the quality of diagnostic accuracy studies [15,16]) to facilitate judgment. In order to obtain a preliminary idea of inter-observer agreement for each item in the RoB tool, Kappa statistics were determined on the basis of 1 systematic review including 32 papers.

## Results

### SYRCLE’s RoB tool

The resulting RoB tool for animal studies contains 10 entries (Table 2). These entries are related to 6 types of bias: selection bias, performance bias, detection bias, attrition bias, reporting bias and other biases. Items 1, 3, 8, 9 and 10 are in agreement with the items in the Cochrane RoB tool. The other items have either been revised or are completely new and will be discussed in greater detail below. Most of the variations between the two tools are a consequence of the differences in design between RCTs and animal studies (see also Table 1). Shortcomings in, or unfamiliarity with, specific aspects of the experimental design of animal studies compared to clinical studies also play a role.

### Bias due to inadequate randomization and lack of blinding

Random allocation of animals to the experimental and control groups, firstly, is not yet standard practice in animal experiments [17]. Furthermore, as the sample size of most animal experiments is relatively small, important baseline differences may be present. Therefore, we propose to include the assessment of similarity in baseline characteristics between the experimental and control groups as a standard item. The number and type of baseline characteristics depend on the review question. Before launching a risk of bias assessment, therefore, reviewers need to discuss which baseline characteristics need to be comparable between the groups.

Secondly, we slightly adjusted the sequence allocation item, specifying that the allocation sequence should not only be adequately generated but also be adequately applied. We decided to do so because, in animal studies, diseases are often induced rather than naturally present. The timing of randomization, therefore, is more important than in a patient setting: it needs to be assessed whether the disease was induced before actual randomization and whether the order of inducement was randomly allocated. The signaling questions for judging this entry are represented in Table 3.

**Table 3:** Signaling questions


*The additional signaling questions are included to assist judgment. “Yes” indicates low risk of bias; “no” indicates high risk of bias; and “unclear” indicates an unclear risk of bias. If one of the relevant signaling questions is answered with “no,” this indicates high risk of bias for that specific entry.*	
**1) Was the allocation sequence adequately generated and applied?**	
*Did the investigators describe a random component in the sequence generation process such as:	Yes/No/Unclear
■ Referring to a random number table;	
■ Using a computer random number generator.	
Additional info:	
Examples of a non-random approach:	
■ Allocation by judgment or by investigator’s preference;	
■ Allocation based on the results of a laboratory test or a series of tests;	
■ Allocation by availability of the intervention;	
■ Sequence generated by odd or even date of birth;	
■ Sequence generated by some rule based on animal number or cage number.	
**2) Were the groups similar at baseline or were they adjusted for confounders in the analysis?**	
*Was the distribution of relevant baseline characteristics balanced for the intervention and control groups?	Yes/No/Unclear
*If relevant, did the investigators adequately adjust for unequal distribution of some relevant baseline characteristics in the analysis?	Yes/No/Unclear
*Was the timing of disease induction adequate?	Yes/No/Unclear
Additional info:	
The number and type of baseline characteristics are dependent on the review question. Before starting their risk of bias assessment, therefore, reviewers need to discuss which baseline characteristics need to be comparable between the groups. In an SR investigating the effects of hypothermia on infarct size, for example, gender distribution, left ventricular weight and heart rate and blood pressure should be similar between the groups at the start of the study.	
A description of baseline characteristics and/or confounders usually contains:	
■ The sex, age and weight of the animals	
■ Baseline values of the outcomes which are of interest in the study	
Timing of disease induction:	
In some prevention studies, the disease is induced after allocation of the intervention. For example, in an experiment on preventive probiotic supplementation in acute pancreatitis, pancreatitis is induced after allocation of the animals to the probiotic or control group. To reduce baseline imbalance, the timing of disease induction should be equal for both treatment groups.	
Examples of adequate timing of disease induction:	
■ The disease was induced before randomization of the intervention.	
■ The disease was induced after randomization of the intervention, but the timing of disease induction was at random, and the individual inducing the disease was adequately blinded from knowing which intervention each animal received.	
**3) Was the allocation to the different groups adequately concealed during?**	
*Could the investigator allocating the animals to intervention or control group not foresee assignment due to one of the following or equivalent methods?	Yes/No/Unclear
■ Third-party coding of experimental and control group allocation Central randomization by a third party	
Sequentially numbered opaque, sealed envelopes	
Additional info:	
Examples of investigators allocating the animals being possibly able to foresee assignments:	
■ Open randomization schedule	
■ Envelopes without appropriate safeguard	
■ Alternation or rotation	
■ Allocation based on date of birth	
■ Allocation based on animal number	
■ Any other explicitly unconcealed procedure of a non-random approach	
**4) Were the animals randomly housed during the experiment?**	
*Did the authors randomly place the cages or animals within the animal room/facility?	Yes/No/Unclear
■ Animals were selected at random during outcome assessment (use signaling questions of entry 6).	
*Is it unlikely that the outcome or the outcome measurement was influenced by not randomly housing the animals?	Yes/No/Unclear
The animals from the various experimental groups live together in one cage/pasture (e.g., housing conditions are identical).	
Additional info:	
Examples of investigators using a non-random approach when placing the cages:	
■ Experimental groups were studied on various locations (e.g., group A in lab A or on shelf A; Group B in Lab B or on shelf B).	
**5) Were the caregivers and/or investigators blinded from knowledge which intervention each animal received during the experiment?**	
*Was blinding of caregivers and investigators ensured, and was it unlikely that their blinding could have been broken?	Yes/No/Unclear
■ ID cards of individual animals, or cage/animal labels are coded and identical in appearance.	
■ Sequentially numbered drug containers are identical in appearance.	
■ The circumstances during the intervention are specified and similar in both groups (#).	
■ Housing conditions of the animals during the experiment are randomized within the room (use criteria of entry 4).	
Additional info:	
Examples of inappropriate blinding:	
■ Colored cage labels (red for group A, yellow group B)	
■ Expected differences in visible effects between control and experimental groups	
■ Housing conditions of the animals are not randomized within the room during the experiment; use criteria of entry 4	
■ The individual who prepares the experiment is the same as the one who conducts and analyses the experiment	
■ Circumstances during the intervention are not similar in both groups (#)	
Examples where circumstances during the intervention were not similar:	
■ Timing of administration of the placebo and exp drug was different.	
■ Instruments used to conduct experiment differ between experimental and control group (e.g., experiment about effects abdominal pressure; exp group receives operation and needle to increase pressure, while control group only has the operation).	
**The relevance of the above-mentioned items depends on the experiment. Authors of the review need to judge for themselves which of the above-mentioned items could cause bias in the results when not similar. These should be assessed.	
**6) Were animals selected at random for outcome assessment?**	
*Did the investigators randomly pick an animal during outcome assessment, or did they use a random component in the sequence generation for outcome assessment?	Yes/No/Unclear
■ Referring to a random number table;	
■ Using a computer random number generator;	
■ Etc.	
**7) Was the outcome assessor blinded?**	
*Was blinding of the outcome assessor ensured, and was it unlikely that blinding could have been broken?	Yes/No/Unclear
■ Outcome assessment methods were the same in both groups.	
■ Animals were selected at random during outcome assessment (use signaling questions of entry 6).	
*Was the outcome assessor not blinded, but do review authors judge that the outcome is not likely to be influenced by lack of blinding?	Yes/No/Unclear
(e.g., mortality)	
Additional info:	
This item needs to be assessed for each main outcome.	
8) Were incomplete outcome data adequately addressed? (*)	
*Were all animals included in the analysis?	Yes/No/Unclear
*Were the reasons for missing outcome data unlikely to be related to true outcome? (e.g., technical failure)	Yes/No/Unclear
*Are missing outcome data balanced in numbers across intervention groups, with similar reasons for missing data across groups?	Yes/No/Unclear
*Are missing outcome data imputed using appropriate methods?	Yes/No/Unclear
9) Are reports of the study free of selective outcome reporting? (*)	
*Was the study protocol available and were all of the study’s pre-specified primary and secondary outcomes reported in the current manuscript?	Yes/No/Unclear
*Was the study protocol not available, but was it clear that the published report included all expected outcomes (i.e. comparing methods and results section)?	Yes/No/Unclear
Additional info:	
Selective outcome reporting:	
- Not all of the study’s pre-specified primary outcomes have been reported;	
- One or more primary outcomes have been reported using measurements, analysis methods or data subsets (e.g., subscales) that were not pre-specified in the protocol;	
- One or more reported primary outcomes were not pre-specified (unless clear justification for their reporting has been provided, such as an unexpected adverse effect);	
- The study report fails to include results for a key outcome that would be expected to have been reported for such a study.	
10) Was the study apparently free of other problems that could result in high risk of bias? (*)	
*Was the study free of contamination (pooling drugs)?	Yes/No/Unclear
*Was the study free of inappropriate influence of funders?	Yes/No/Unclear
*Was the study free of unit of analysis errors?	Yes/No/Unclear
*Were design-specific risks of bias absent?	Yes/No/Unclear
*Were new animals added to the control and experimental groups to replace drop-outs from the original population?	Yes/No/Unclear
Additional info:	
The relevance of the signaling questions (Table 3) depends on the experiment. Review authors need to judge for themselves which of the items could cause bias in their results and should be assessed.	
Contamination/pooling drugs:	
Experiments in which animals receive ‒ besides the intervention drug ‒ additional treatment or drugs which might influence or bias the result.	
Unit of analysis errors:	
■ Interventions to parts of the body within one participant (i. e., one eye exp; one eye control).	
■ All animals receiving the same intervention are caged together, but analysis was conducted as if every single animal was one experimental unit.	
Design-specific risks of bias:	
■ Crossover design that was not suitable (intervention with no temporary effect, or the disease is not stable over time)	
■ Crossover design with risk of carry-over effect	
■ Crossover design with only first period data being available	
■ Crossover design with many animals not receiving 2^nd^ or following treatment due to large number of drop-outs probably due to longer duration of study	
■ Crossover design in which all animals received same order of interventions	
■ Multi-arm study in which the same comparisons of groups are not reported for all outcomes (selective outcome reporting)	
■ Multi-arm study in which results of different arms are combined (all data should be presented per group)	
■ Cluster randomized trial not taking clustering into account during statistical analysis (unit of analysis error)	
■ Crossover design in which paired analysis of the results is not taken into account	

Thirdly, a new item pertains to randomizing the housing conditions of animals during the experiment. In animal studies, the investigators are responsible for the way the animals are housed. They determine, for example, the location of the cage in the room. As housing conditions (such as lighting, humidity, temperature, etc.) are known to influence study outcomes (such as certain biochemical parameters and behavior), it is important that the housing of these animals is randomized or, in other words, comparable between the experimental groups in order to reduce bias [18]. Animals from different treatment groups, for example, should not be housed per group on different shelves or in different rooms as the animals on the top shelf experience a higher room temperature than animals on the lowest shelf, and the temperature of the room may influence the toxicity of pharmacological agents (Table 4). When cages are not placed randomly (e.g., when animals are housed per group on different shelves), moreover, it is possible for the investigator to foresee or predict the allocation of the animals to the various groups, which might result in performance bias. Therefore, randomizing the housing conditions is also a requisite for adequately blinding the animal caregivers and investigators. Therefore, this has also been included as a signaling question in Table 3.

**Table 4:** Some underlying evidence for the importance of random housing and random outcome assessment

**Random housing**		
Lighting	Light exposure varies with respect to rack location and position of cages within the rack	[27,28]
	Small differences in light intensity have been associated with reproductive and behavioral changes	[27,29,30]
	There can be a four-fold difference in light intensity between cages at the top or bottom of a rack	[18]
Temperature	Temperature in animal room at 1.5 m can be 3-4˚C higher than at 0.5 m	[18]
	Cage temperature varies with group size	[18]
	Cage temperature varies with height of placement within the rack (top rack 5˚C warmer than bottom rack)	[18,31,32]
	Small changes in temperature can influence metabolic rates and toxicity	[27,31,33]
**Random outcome assessment**		
Circadian rhythm	Periodic/circadian variations in lipid metabolism, neurotransmitter levels, pharmacokinetic effects, etc.	[34-37]

Suggestions for further reading: [18,27,37,38].

Fourthly, in a recent update of the Cochrane RoB tool (http://www.cochrane.org/sites/default/files/uploads/handbook/Whats%20new%20in%20Handbook%205_1_0.pdf), bias related to blinding of participants and personnel (performance bias) is assessed separately from bias related to blinding of outcome assessment (detection bias). In our tool, we followed this approach, although animals do not need to be blinded for the intervention as they do not have any expectations about the intervention. In addition, it is important to emphasize that personnel involved in the experimental animal studies should be taken to include animal caregivers. In animal studies, this group is often not taken into account when blinding the allocation of animals to various groups. If animal caregivers know that a drug might cause epileptic seizures or increases urine production, for example, they might handle the animals or clean the cages in the group receiving this drug more often, which could cause behavioral changes influencing the study results.

With regard to adequately blinding outcome assessment (entry 7), possible differences between the experimental and control groups in methods used for outcome assessment should be described and judged. It should also be determined whether or not animals were selected at random for outcome assessment, regardless of the allocation to the experimental or control group. For instance, when animals are sacrificed per group at various time points during the day, the scientist concerned might interpret the results of the groups differently because she or he can foresee or predict the allocation.

Another reason to select animals at random for outcome assessment is the presence of circadian rhythms in many biological processes (Table 4). Not selecting the animals for outcome assessment at random might influence the direction and magnitude of the effect. For example, the results of a variety of blood tests depend on their timing during the day: cholesterol levels in mice may be much higher in the morning after a meal than in the afternoon. Because of these effects, assessing whether or not animals were selected at random for outcome assessment has also been presented as a separate entry.

#### ***Reporting bias***

As mentioned before, assessing reporting bias is in agreement with the Cochrane RoB tool. It is important to mention, however, that this item is quite difficult to assess in animal intervention studies at present because protocols for animal studies are not yet registered in a central, publicly accessible database. Nevertheless, many have called for registration of all animal experiments at inception [19,20], so we expect that registration of animal studies will be more common within a few years. For this reason, we already decided to include it in SYRCLE’s RoB tool. Furthermore, protocols of animal studies, like those of clinical studies, can already be published in various (open access) journals, which will also help to improve the standard of research in animal sciences.

#### ***Other bias***

Beyond the above-mentioned types of bias, there might be further issues that may raise concerns about the possibility of bias. These issues have been summarized in the other bias domain. The relevance of the signaling questions (Table 3) depends on the experiment. Review authors need to judge for themselves which of the items could cause bias in their results and should be assessed. In assessing entry 10 (“Was the study apparently free of other risks of bias?”), it is important to pay extra attention to the presence of unit-of-analysis errors. In animal studies, the experimental unit is often not clear, and as a consequence statistical measures are often inaccurately calculated. For example, if mice in a cage are given a treatment in their diet, it is the cage of animals rather than the individual animal that is the experimental unit. After all, the mice in the cage cannot have different treatments, and they may be more similar than mice in different cages.

### Use of SYRCLE’s RoB tool

In order to assign a judgment of low, high or unclear risk of bias to each item mentioned in the tool, we have produced a detailed list with signaling questions to aid the judgment process (Table 3). It is important to emphasize that this list is not exhaustive. We recommend that people assessing the risk of bias of the included studies discuss and adapt this list to the specific needs of their review in advance. A “yes” judgement indicates a low risk of bias; a “no” judgment indicates high risk of bias; the judgment will be “unclear” if insufficient details have been reported to assess the risk of bias properly.

As a rule, assessments should be done by at least two independent reviewers, and disagreements should be resolved through consensus-oriented discussion or by consulting a third person.

We recommend that risk of bias assessment is presented in a table or figure. The investigators can present either the summary results of the risk of bias assessment or the results of all individual studies. Finally, the results of the risk of bias assessment could be used when interpreting the results of the review or a meta-analysis. For instance, sensitivity analysis can be used to show how the conclusions of the review might be affected if studies with a high risk of bias were excluded from the analysis [8,9].

We do not recommend calculating a summary score for each individual study when using this tool. A summary score inevitably involves assigning “weights” to specific domains in the tool, and it is difficult to justify the weights assigned. In addition, these weights might differ per outcome and per review.

### Inter-observer variability

Inter-observer agreement was evaluated using Kappa statistics. At time of writing, the Kappa statistics could only be determined for items 1, 6, 7, 8, 9 and 10 and was based on 2 raters in one systematic review including 32 papers. For items 1, 6, 7, 8, 9 and 10, the inter-observer variability varied between 0.62 and 1.0. Kappa was for item 1: 0.87; item 6: 0.74; item 7: 0.59; item 8: 1.0; item 9: 0.62; item 10: 1.0. Kappa could not be calculated for items 2, 3, 4, and 5 as Kappa is defined for situations with at least two raters and two outcomes, and in these items we had only 1 outcome (unclear risk of bias) as a result of poor reporting.

## Discussion and conclusion

In animal studies, a large variety of tools to assess study quality is currently used, but none of the tools identified so far focussed on internal validity only [11]. Most instruments assess reporting quality and internal and external validity simultaneously although consequences of poor reporting, risk of bias and generalizability of the results are very different.

Therefore, we developed SYRCLE’s RoB tool to establish consistency and avoid discrepancies in assessing risk of bias in SRs of animal intervention studies. SYRCLE’s RoB tool is based on the Cochrane RoB tool [9] and has been adjusted for particular aspects of bias that play a role in animal intervention studies. All items in our RoB tool can be justified from a theoretical perspective, but not all items have been validated by empirical research. However, the same holds for the original QUADAS tool (to assess the quality of diagnostic accuracy studies) and the Cochrane RoB tool [8,16]. For example, in the Cochrane RoB tool, the item on “inadequately addressing incomplete outcome data” is mainly driven by theoretical considerations [8]. In QUADAS, no empirical or theoretical evidence was available for 2 out of the 9 risk of bias items [16].

Although validation is important, providing empirical evidence for all items in this tool is not to be expected in the near future as this would require major comparative studies, which, to our knowledge, are not currently being undertaken or scheduled. Using the existing animal experimental literature is also challenging because the current reporting quality of animal studies is poor [17]; many details regarding housing conditions or timing outcome assessment are often unreported. However, we feel that publishing this tool is necessary to increase awareness of the importance of improving the internal validity of animal studies and to gather practical experience of authors using this tool.

We started to use this tool in our own SRs and hands-on training courses on conducting SRs in laboratory animal experimentation, funded by The Netherlands Organization for Health Research and Development (ZonMW). The first experiences with this tool were positive, and users found SYRCLE’s RoB tool very useful. The inter-rater variability Kappa varied between 0.6 and 1 9. Users also indicated that they had to judge many entries as “unclear risk of bias”. Although most users did not expect this finding, it is not altogether surprising [21,22], as a recent survey of 271 animal studies revealed that reporting experimental details on animals, methods and materials is very poor [17]. We hope and expect, therefore, that use of this tool will improve the reporting quality of essential experimental details in animal studies [23,24].

Widespread adoption and implementation of this tool will facilitate and improve critical appraisal of evidence from animal studies. This may subsequently enhance the efficiency of translating animal research results into clinical practice. Furthermore, this tool should be tested by authors of SRs of animal intervention studies to test its applicability and validity in practice. We invite users of SYRCLEs RoB tool, therefore, to provide comments and feedback via the SYRCLE LinkedIn group (risk of bias subgroup) http://www.linkedin.com/groups?gid=4301693&trk=hb_side_g. As with the QUADAS, CONSORT and PRISMA statements [15,16,25,26], we expect that user feedback and developments in this relatively new field of evidence-based animal experimentation will allow us to update this tool within a few years.

## Competing interests

All authors declare that none of the authors (Hooijmans, Rovers, de Vries, Leenaars, Ritskes-Hoitinga, Langendam) have anything to disclose or any competing interests. The authors had no support from any organization for the submitted work; no financial relationships with any organizations that might have an interest in the submitted work in the previous three years, no other relationships or activities that could appear to have influenced the submitted work.

## Authors’ contributions

CRH coordinated the project. CRH, MWL and MMR have made substantial contributions to the design of the RoB tool, article conception and wrote the first draft of the paper. MRH, ML and RdV provided advice on bias in animal studies (part of the discussion group). MRH and RdV revised the manuscript. All authors read and approved the final manuscript.

## Pre-publication history

The pre-publication history for this paper can be accessed here:

http://www.biomedcentral.com/1471-2288/14/43/prepub

## Supplementary Material

Additional file 1 **A pilot survey to provide some supportive information for some of the statements made in Table **1**.** Click here for file
