# Supplementary material for: SYRCLE’s risk of bias tool for animal studies
Source: BMC Med Res Methodol. 2014 Mar 26;14:43. doi: 10.1186/1471-2288-14-43 (PMC4230647; doi:10.1186/1471-2288-14-43)
Supplement: Additional file 1 — A pilot survey to provide some supportive information for some of the statements made in Table 1. [file 1471-2288-14-43-S1.docx]

**Additional file 1:**

A small pilot survey was conducted to provide additional support for some of the statements made in table 1.

Methods:

Selecting Cochrane reviews

The Cochrane Library was sorted by “new reviews”. The first 15 recent Cochrane reviews describing an intervention or clinical efficacy were selected. Reviews in which no RCTs could be included were excluded from this survey.

Selecting SRs of animal experiments

Pubmed was searched for all SRs of animal studies in 2013 (search strategy see below). All SRs describing the efficacy of an intervention for human health care were selected. From the resulting 116 SRs, every seventh SR was screened full text and included when the nr of animals and nr of included studies were clearly described.

Search strategy

(systematic review[ti] OR systematic reviews[ti] OR meta-analysis[pt] OR meta-analyses[ti] OR meta-analysis[ti] OR systematic literature review[ti] OR (systematic review[tiab] AND review[pt]) OR Systematic survey[tiab] OR Critical survey[tiab] OR Systematic surveys[tiab] OR (systematic overview[tiab] OR systematic overviews[tiab]) OR critical overview[tiab] OR systematic review[tiab] OR systematically review[tiab] OR critical review[tiab] OR systematic reviews[tiab] OR critical reviews[tiab]) NOT (letter[pt] OR newspaper article[pt] OR comment[pt]) Filters: Review; Systematic Reviews; Publication date from 2013/01/01 to 2013/12/01

Outcome assessment:

For both the Cochrane SRs and SRs of animal studies, the number of included studies and number of included subjects were assessed.

For Cochrane SRs, the risk of bias analyses were checked for random allocation, allocation concealment and blinding of the outcome assessment.

For animal studies, this was not possible as most SRs of animal studies do not yet conduct a risk of bias analysis, and a large proportion of the animal studies do not report about randomization or blinding at all.

Results:

15 Cochrane SRs and 13 SRs of animal studies (extracted from 12 publications) were analysed. These publications include in total 125 RCTs and 455 animal experiments/ comparisons.

Sample size:

The average sample size of the 125 included RCTs was 201 patients. The average sample size of 455 animal experiments (extracted from 13 SRs) was 19. This is a factor 10 difference.

This supports our statement that sample sizes of RCTs are in general larger than sample size of animal studies.

Internal validity:

Our pilot survey showed that adequate random allocation occurred in 36% of the RCTs. Adequate allocation concealment occurred in 29.6% of the RCTs, and adequate blinding of the outcome assessment in 33.6% of the RCTs.

We could, however, not assess this in SRs of animal studies, because reporting of essential details in animal studies is very poor. In addition, when these items are assessed in animal studies criteria for judging adequate randomisation or blinding are very different from the criteria used in clinical trials.

| References (Cochrane reviews) | 1 | 2 | 3 | 4 | 5 | 6 | 7 | 8 | 9 | 10 | 11 | 12 | 13 | 14 | 15 | Total | % of total # of studies |
| --- | --- | --- | --- | --- | --- | --- | --- | --- | --- | --- | --- | --- | --- | --- | --- | --- | --- |
| # of studies included in review | 7 | 3 | 2 | 4 | 12 | 13 | 3 | 3 | 6 | 1 | 9 | 44 | 5 | 11 | 2 | 125 |  |
| # of subjects | 2241 | 102 | 4771 | 199 | 2317 | 4495 | 47 | 213 | 278 | 306 | 622 | 6580 | 203 | 2678 | 98 | 25150 |  |
| # of studies adequate random allocation | 5 | 1 | 2 | 2 | 3 | 5 | 1 | 0 | 1 | 1 | 2 | 15 | 0 | 7 | 0 | 45 | 36 |
| # of studies unclear random allocation | 1 | 1 | 0 | 2 | 9 | 8 | 2 | 3 | 5 | 0 | 7 | 24 | 5 | 2 | 2 | 71 | 56.8 |
| # of studies high risk random allocation | 1 | 1 | 0 | 0 | 0 | 0 | 0 | 0 | 0 | 0 | 0 | 5 | 0 | 2 | 0 | 9 | 7.2 |
| # of studies adequate allocation concealment | 3 | 1 | 2 | 3 | 2 | 4 | 1 | 2 | 0 | 0 | 4 | 11 | 1 | 3 | 0 | 37 | 29.6 |
| # of studies unclear allocation concealment | 1 | 1 | 0 | 1 | 9 | 9 | 2 | 1 | 6 | 1 | 5 | 29 | 4 | 7 | 2 | 78 | 62.4 |
| # of studies high risk allocation concealment | 3 | 1 | 0 | 0 | 1 | 0 | 0 | 0 | 0 | 0 | 0 | 4 | 0 | 1 | 0 | 10 | 8 |
| # of studies adequate blinding outcome assessment | 3 | 0 | 0 | 3 | 2 | 12 | 3 | 2 | 0 | 0 | 6 | 8 | 2 | 1 | 0 | 42 | 33.6 |
| # of studies unclear blinding outcome assessment | 4 | 3 | 0 | 4 | 10 | 0 | 0 | 0 | 4 | 0 | 3 | 21 | 3 | 10 | 2 | 64 | 51.2 |
| # of studies high risk blinding outcome assessment | 0 | 0 | 2 | 0 | 0 | 1 | 0 | 1 | 2 | 1 | 0 | 15 | 0 | 0 | 0 | 22 | 17.6 |
|  |  |  |  |  |  |  |  |  |  |  |  |  |  |  |  |  |  |
| References (SRs animal studies) | 16 | 17 | 17 | 18 | 19 | 20 | 21 | 22 | 23 | 24 | 25 | 26 | 27 |  |  | 455 |  |
| # of experiments included in review | 9 | 14 | 11 | 20 | 12 | 149 | 17 | 9 | 43 | 50 | 75 | 37 | 9 |  |  | 8828 |  |
| # of subjects | 63 | 241 | 207 | 1562 | 189 | 2443 | 863 | 136 | 448 | 880 | 1270 | 414 | 112 |  |  |  |  |

References

[[1-16](#_ENREF_1)] [[17](#_ENREF_17)] [[18-26](#_ENREF_18)] [[27](#_ENREF_27)]

1. Bailey E, Worthington HV, van Wijk A, Yates JM, Coulthard P, Afzal Z: **Ibuprofen and/or paracetamol (acetaminophen) for pain relief after surgical removal of lower wisdom teeth**. *The Cochrane database of systematic reviews* 2013, **12**:CD004624.

2. Basuki F, Hadiati DR, Turner T, McDonald S, Hakimi M: **Dilute versus full strength formula in exclusively formula-fed preterm or low birth weight infants**. *The Cochrane database of systematic reviews* 2013, **11**:CD007263.

3. Brocklehurst P, Price J, Glenny AM, Tickle M, Birch S, Mertz E, Grytten J: **The effect of different methods of remuneration on the behaviour of primary care dentists**. *The Cochrane database of systematic reviews* 2013, **11**:CD009853.

4. Cox NS, Alison JA, Holland AE: **Interventions for promoting physical activity in people with cystic fibrosis**. *The Cochrane database of systematic reviews* 2013, **12**:CD009448.

5. Dear RF, McGeechan K, Jenkins MC, Barratt A, Tattersall MH, Wilcken N: **Combination versus sequential single agent chemotherapy for metastatic breast cancer**. *The Cochrane database of systematic reviews* 2013, **12**:CD008792.

6. Guaiana G, Gupta S, Chiodo D, Davies SJ, Haederle K, Koesters M: **Agomelatine versus other antidepressive agents for major depression**. *The Cochrane database of systematic reviews* 2013, **12**:CD008851.

7. Gurusamy KS, Koti R, Toon CD, Wilson P, Davidson BR: **Antibiotic therapy for the treatment of methicillin-resistant Staphylococcus aureus (MRSA) in non surgical wounds**. *The Cochrane database of systematic reviews* 2013, **11**:CD010427.

8. Kong X, Yuan H, Fan J, Li Z, Wu T, Jiang L: **Lipid-lowering agents for nephrotic syndrome**. *The Cochrane database of systematic reviews* 2013, **12**:CD005425.

9. Okusanya BO, Oladapo OT: **Prophylactic versus selective blood transfusion for sickle cell disease in pregnancy**. *The Cochrane database of systematic reviews* 2013, **12**:CD010378.

10. Peckham EJ, Nelson EA, Greenhalgh J, Cooper K, Roberts ER, Agrawal A: **Homeopathy for treatment of irritable bowel syndrome**. *The Cochrane database of systematic reviews* 2013, **11**:CD009710.

11. Penninga L, Moller CH, Penninga EI, Iversen M, Gluud C, Steinbruchel DA: **Antibody induction therapy for lung transplant recipients**. *The Cochrane database of systematic reviews* 2013, **11**:CD008927.

12. Perry AE, Neilson M, Martyn-St James M, Glanville JM, McCool R, Duffy S, Godfrey C, Hewitt C: **Pharmacological interventions for drug-using offenders**. *The Cochrane database of systematic reviews* 2013, **12**:CD010862.

13. Reilly S, Planner C, Gask L, Hann M, Knowles S, Druss B, Lester H: **Collaborative care approaches for people with severe mental illness**. *The Cochrane database of systematic reviews* 2013, **11**:CD009531.

14. Tian H, Guo X, Wang X, He Z, Sun R, Ge S, Zhang Z: **Chromium picolinate supplementation for overweight or obese adults**. *The Cochrane database of systematic reviews* 2013, **11**:CD010063.

15. Verhagen AP, Bierma-Zeinstra SM, Burdorf A, Stynes SM, de Vet HC, Koes BW: **Conservative interventions for treating work-related complaints of the arm, neck or shoulder in adults**. *The Cochrane database of systematic reviews* 2013, **12**:CD008742.

16. Alves M, Jr., Baratieri C, Mattos CT, Araujo MT, Maia LC: **Root repair after contact with mini-implants: systematic review of the literature**. *European journal of orthodontics* 2013:491-499.

17. Batchelor PE, Skeers P, Antonic A, Wills TE, Howells DW, Macleod MR, Sena ES: **Systematic review and meta-analysis of therapeutic hypothermia in animal models of spinal cord injury**. *PloS one* 2013:e71317.

18. Fritz H, Seely D, Kennedy DA, Fernandes R, Cooley K, Fergusson D: **Green tea and lung cancer: a systematic review**. *Integrative cancer therapies* 2013:7-24.

19. Hamilton N, Bullock AJ, Macneil S, Janes SM, Birchall M: **Tissue engineering airway mucosa: A systematic review**. *The Laryngoscope* 2013.

20. Hirst TC, Vesterinen HM, Sena ES, Egan KJ, Macleod MR, Whittle IR: **Systematic review and meta-analysis of temozolomide in animal models of glioma: was clinical efficacy predicted?** *British journal of cancer* 2013:64-71.

21. Knapik DM, Harris JD, Pangrazzi G, Griesser MJ, Siston RA, Agarwal S, Flanigan DC: **The basic science of continuous passive motion in promoting knee health: a systematic review of studies in a rabbit model**. *Arthroscopy : the journal of arthroscopic & related surgery : official publication of the Arthroscopy Association of North America and the International Arthroscopy Association* 2013:1722-1731.

22. Lui PP, Ng SW: **Cell therapy for the treatment of tendinopathy--a systematic review on the pre-clinical and clinical evidence**. *Seminars in arthritis and rheumatism* 2013:651-666.

23. Oudman I, Clark JF, Brewster LM: **The effect of the creatine analogue beta-guanidinopropionic acid on energy metabolism: a systematic review**. *PloS one* 2013:e52879.

24. Schmidt A, Wellmann J, Schilling M, Strecker JK, Sommer C, Schabitz WR, Diederich K, Minnerup J: **Meta-analysis of the Efficacy of Different Training Strategies in Animal Models of Ischemic Stroke**. *Stroke; a journal of cerebral circulation* 2013.

25. Wei RL, Teng HJ, Yin B, Xu Y, Du Y, He FP, Chu KT, Luo BY, Zheng GQ: **A systematic review and meta-analysis of buyang huanwu decoction in animal model of focal cerebral ischemia**. *Evidence-based complementary and alternative medicine : eCAM* 2013:138484.

26. Yen CC, Tu YK, Chen TH, Lu HK: **Comparison of treatment effects of guided tissue regeneration on infrabony lesions between animal and human studies: a systematic review and meta-analysis**. *Journal of periodontal research* 2013.

27. Luks V, Burkett A, Turner L, Pakhale S: **Effect of physical training on airway inflammation in animal models of asthma: a systematic review**. *BMC pulmonary medicine* 2013:24.
